# Comparison of a novel chemiluminescent based algorithm to three algorithmic approaches for the laboratory diagnosis of *Clostridium difficile* infection

**DOI:** 10.1186/s13099-015-0079-8

**Published:** 2015-12-23

**Authors:** J. Goret, J. Blanchi, C. Eckert, S. Lacome, A. Petit, F. Barbut, C. Bébéar, Francis Mégraud

**Affiliations:** Laboratoire de Bactériologie, C.H.U. de Bordeaux, Hôspital Pellegrin, 33076 Bordeaux Cedex, France; National Reference Laboratory for Clostridium difficile, Assistance Publique-Hôpitaux de Paris, Hôpital Saint-Antoine, Paris, France

**Keywords:** *Clostridium difficile*, Algorithm, Chemiluminescence, Diagnosis, Toxins, DiaSorin

## Abstract

**Background:**

Rapid commercial assays, including nucleic acid amplification tests and immunoassays for *Clostridium. difficile* toxins, have replaced the use of older assays. They are included in a two-step algorithm diagnosis, including first the detection of the glutamate dehydrogenase (GDH) as a screening method and second the detection of toxins as a confirmatory method. Although assays that detect the presence of free toxins in feces are known to lack sensitivity, they are preferable to confirm infection. We evaluated the accuracy of the chemiluminescence-based method detecting *C.difficile* GDH and free toxins A/B (DiaSorin algorithm) to an enzyme-immunoassay (EIA) for GDH with a molecular toxins test (Meridian algorithm), EIA-GDH and an EIA-toxins A/B algorithm (Alere algorithm) with and without toxigenic culture for confirmation.

**Findings:**

A total of 468 diarrhoeal and loose stool samples were included in the study. A positive result was defined by a positive GDH and a positive toxin test. Discordant samples were resolved using an enriched toxigenic culture considered as the reference method. After resolution, the DiaSorin algorithm showed a high sensitivity (86.7 %) compared to that of the Alere algorithm with (60.0 %) and without (50.0 %) confirmation by culture and was as sensitive as the Meridian algorithm (90.0 %), while the specificities were similar: 99.1, 99.5, 99.5 and 98.9 %, respectively.

**Conclusions:**

The DiaSorin algorithm was as sensitive as an algorithm including nucleic acid amplification test for toxins. Chemiluminescence toxin-enhanced signal assay compensates the lack of sensitivity usually observed for EIA tests for toxins.

## Background

*Clostridium difficile* is a major cause of healthcare-associated diarrhoea. Many different approaches are available for the laboratory diagnosis of *C*. *difficile* infection (CDI). American [1] and European [2] guidelines recommend testing patients with a two-step algorithm including the detection of glutamate dehydrogenase (GDH) as a screening method followed, in case of positive result, by the detection of free toxins or their genes as a confirmation method. The DiaSorin Liaison^®^*C.difficile* GDH and toxins A and B (DiaSorin, Saluggia, Italy) is a sandwich chemiluminescent immunoassay (CLIA) performed on a stool extract. Luminescent assays for *C.difficile* diagnosis have not been previously reported in the literature. This method may be more reliable than enzyme immunoassays (EIA), which are known to lack sensitivity for free toxin detection. The objective of the study was to evaluate the performances of the newly available chemiluminescence-based DiaSorin algorithm for the detection of GDH and free toxins A and B using the Liaison apparatus in a routine laboratory.

The DiaSorin algorithm was compared to three other algorithms: (1) a widely used EIA algorithm including, the C. Diff Quik Chek GDH^®^ followed by detection of free toxins A and B, TOX A/B Quik Chek^®^ test (Alere, Waltham, MA, USA), (2) the same EIA-based algorithm with toxigenic culture as a third step in case of negative toxin results; for this purpose, the stools were directly inoculated on a cycloserine-cefoxitin-amphotericin agar (bioMérieux, Marcy l’Etoile, France) incubated at 37 °C for 3 days in an anaerobic atmosphere and the isolates were then tested for toxins A and B using the TOX A/B Quik Chek^®^ (Alere), and (3) a very sensitive algorithm [3–8] using the EIA ImmunoCard^®^*C.difficile* GDH for screening followed by a loop-mediated isothermal amplification assay for *tcdA* toxin gene detection (*illumigene*^®^ Meridian, Cincinnati, OH, USA). The manufacturer’s recommendations were followed. All algorithms included a preliminary screening step for the detection of GDH and then, if positive, the stool samples were tested for toxins or toxin genes. The specimens that tested positive for the toxins were considered positive. The specimens that tested negative for GDH or for toxins were considered negative. In our study, the aggregate criteria for a true positive or a true negative result were a positive or negative result for the four algorithms, respectively. If an algorithm result was different from the three others, the sample was considered discordant. The discordant samples were resolved using enriched toxigenic culture (ETC) considered as a reference method and performed at the French National Reference Laboratory for *C.difficile* (Paris, France): stool samples were inoculated in pre-reduced taurocholate-cycloserine-cefoxitin BHI broth incubated for 5 days at 37 °C under anaerobic conditions; the broth was then plated onto laboratory standard selective plates containing taurocholate, cycloserine and cefoxitin. Toxinogenicity of the strains was demonstrated using an in-house polymerase chain reaction (PCR) targeting *tcd*A and *tcd*B genes. After ETC, a stool sample was considered positive for toxigenic *C.difficile* if the toxin genes were detected.

Between June and September 2013, *C.difficile* testing was prospectively performed with the four different algorithms at the Bacteriology Laboratory of the Bordeaux University Hospital (Bordeaux, France) on diarrhoeal stools either upon a physician’s request or systematically inpatients with diarrhea after day 3 of hospitalization. As recommended in the literature [9] and manufacturers’ instructions, the followings samples were excluded: formed stools, bloody stools, stools submitted from a patient with a positive *C.difficile* test result in the preceding 7 days, stool samples which were received in the laboratory more than 48 h after emission, and samples from patients less than 2 years of age. A total of 468 stool samples were included in the study. Before resolution, the overall algorithm concordance was 94.7 % (443/468) with 2.8 % true positive results (13/468), 91.9 % true negative results (430/468), and 5.3 % (25/468) discordant results. The 25 discordant samples were subjected to ETC (Table 1). Among the discordant samples, the DiaSorin algorithm showed 13 true positive, 4 true negative, 4 false positive and 4 false negative results after resolution. The sensitivity, specificity, positive and negative predictive values of the DiaSorin Liaison were 86.7, 99.1, 86.7 and 98.1 % (Table 1), respectively. There was no significant difference for sensitivity and specificity between the DiaSorin and Meridian algorithm (90.0 %) using a molecular test (McNemar’s test, p = 0.72). Moreover, the DiaSorin algorithm exhibited a significantly higher sensitivity compared to that of Alere with toxigenic culture confirmation (60.0 %) and without confirmation (50.0 %). There was no significant difference in GDH results between the screening assays (CLIA and EIA): the overall concordance for GDH tests was 95.7 % with 15 % of positive results.

**Table 1:** *Clostridium difficile* diagnostic test performances using enriched toxigenic culture as the reference standard

	(%) Sensitivity (95% CI)	(%) Specificity (95% CI)	Positive predictive value (%) (95% CI)	Negative predictive value (%) (95% CI)
DiaSorin algorithm = CLIA GDH + CLIA toxins A/B	86.7 (68.4–95.6)	99.1 (97.7–99.8)	86.7 (68.3–95.6)	98.1 (97.5–99.7)
Meridian algorithm = EIA GDH + NAAT for toxin gene	90.0 (72.3–97.8)	98.9 (97.4–99.5)	84.4 (66.5–94.1)	99.3 (97.8–99.8)
2-Step Alere algorithm = EIA GDH + EIA toxins A/B	50.0 (31.7–68.3)	99.5 (98.1–99.9)	88.2 (62.2–97.9)	96.7 (94.5–98.1)
3-Step Alere algorithm = EIA GDH + EIA toxins A/B + toxigenic culture	60.0 (40.7–76.7)	99.5 (98.1–99.9)	90.0 (66.8–98.2)	97.3 (95.2–98.5)

*CLIA* chemiluminescence, *GDH* glutamate dehydrogenase, *EIA* enzyme immunoassay, *NAAT* nucleic acid amplification test, *CI* 95 % confidence interval

Since the algorithms were based on an initial GDH screening with no significantly different results observed, their performance was related to the part of the test confirming the toxins. As expected, the novel DiaSorin chemiluminescent algorithm has a high sensitivity indicating that this sandwich ELISA-like test with enhanced signaling indeed compensates for the lack of sensitivity usually observed with EIA-based assays. Surprisingly, the overall performance of the DiaSorin algorithm was as high as the Meridian algorithm, already known to be very accurate. Combining this two-step algorithm with a confirmation test was not required. Moreover, tests that detect the presence of free *C.difficile* toxins in feces are significantly associated with clinical outcomes [10]. A retrospective chart review was performed for all the positive results of the DiaSorin algorithm. There was a good correlation with clinical pictures (Table 2).

**Table 2:** Turnaround time and reagent costs to detect *Clostridium difficile* infection in the laboratory

		Stool extraction	Labor time (min)	Turnaround time of test (min)	Apparatus	Individual or series test	Algorithm cost ratio relative to DiaSorin algorithm (%)
DiaSorin	GDH	Yes	15	45	Liaison random access	Both	100
	Toxins A and B	Yes^a^	0^a^	45	Liaison random access	Both	
Meridian	ImmunoCard^®^ GDH	No	5	30	No	Individual	90
	*illumigene* ^*®*^	No	15	45	illumipro^®^	Individual	
Alere	C. DIFF Quik Chek GDH^®^	No	5	30	No	Individual	50
	TOX A/B Quik Chek^®^	No	5	30	No	Individual	

The cost of each algorithm was evaluated in comparison to the DiaSorin algorithm whose the global costs represent 100 %

^a^The DiaSorin LIAISON^®^ apparatus is configured in order to automatically perform the toxin assay in case of a positive GDH result (reflex testing). These two assays were performed on the same stool extract

The limitation of the study was the small number of positive cases rendering the estimation of sensitivity with a large confidence interval. Given the low incidence of CDI in this population, a larger number of specimens should have been evaluated. Choosing the right diagnostic approach is a matter of test accuracy, turnaround time, and cost for routine use in a clinical laboratory. In conclusion there is a good performance of the DiaSorin assay in comparison to the three other approaches. This method is sufficient for the diagnosis of CDI and there is no need for a confirmation test because the sensitivity of the test is as high as the molecular method. This algorithm could replace the others.
